# MAPK/ERK-PK(Ser11) pathway regulates divergent thermal metabolism of two congeneric oyster species

**DOI:** 10.1016/j.isci.2024.110321

**Published:** 2024-06-20

**Authors:** Chaogang Wang, Mingyang Du, Zhuxiang Jiang, Rihao Cong, Wei Wang, Taiping Zhang, Jincheng Chen, Guofan Zhang, Li Li

**Affiliations:** 1 CAS and Shandong Province Key Laboratory of Experimental Marine Biology, Center for Ocean Mega-Science, Institute of Oceanology, Chinese Academy of Sciences, Qingdao, China; 2 Laboratory for Marine Biology and Biotechnology, Qingdao Marine Science and Technology Center, Qingdao, Shandong, China; 3 University of Chinese Academy of Sciences, Beijing, China; 4 Key Laboratory of Breeding Biotechnology and Sustainable Aquaculture, Institute of Hydrobiology, Chinese Academy of Sciences, Wuhan, China; 5 Laboratory for Marine Fisheries Science and Food Production Processes, Qingdao Marine Science and Technology Center, Qingdao, Shandong, China; 6 National and Local Joint Engineering Laboratory of Ecological Mariculture, Institute of Oceanology, Chinese Academy of Sciences, Qingdao, China; 7 Shandong Technology Innovation Center of Oyster Seed Industry, Qingdao, China; 8 Southern Marine Science and Engineering Guangdong Laboratory (Zhanjiang), Zhanjiang, China

**Keywords:** Zoology, Molecular biology, Evolutionary biology

## Abstract

Pyruvate kinase (PK), as a key rate-limiting enzyme in glycolysis, has been widely used to assess the stress tolerance and sensitivity of organisms. However, its phosphorylation regulatory mechanisms mainly focused on human cancer research, with no reports in marine organisms. In this study, we firstly reported a conserved PK Ser11 phosphorylation site in mollusks, which enhanced enzyme activity by promoting substrate binding, thereby regulating divergent thermal metabolism of two allopatric congeneric oyster species with differential habitat temperature. It was phosphorylated by ERK kinase, and regulated by the classical MAPK pathway. The MAPK/ERK-PK signaling cascade responded to increased environmental temperature and exhibited stronger activation pattern in the relatively thermotolerant species (*Crassostrea angulata*), indicating its involvement in shaping temperature adaptation. These findings highlight the presence of complex and unique phosphorylation-mediated signaling transduction mechanisms in marine organisms, and provide new insights into the evolution and function of the crosstalk between classical pathways.

## Highlights

• The Ser11 site of PK is conserved in Gastropoda and Bivalvia
• Phosphorylation of Ser11 site of PK significantly enhances its enzymatic activity
• MAPK/ERK1/2 phosphorylates the Ser11 site of PK under heat stress
• MAPK/ERK-PK cascade mediates divergent thermal metabolism capacity

## Graphical abstract

**Figure:**
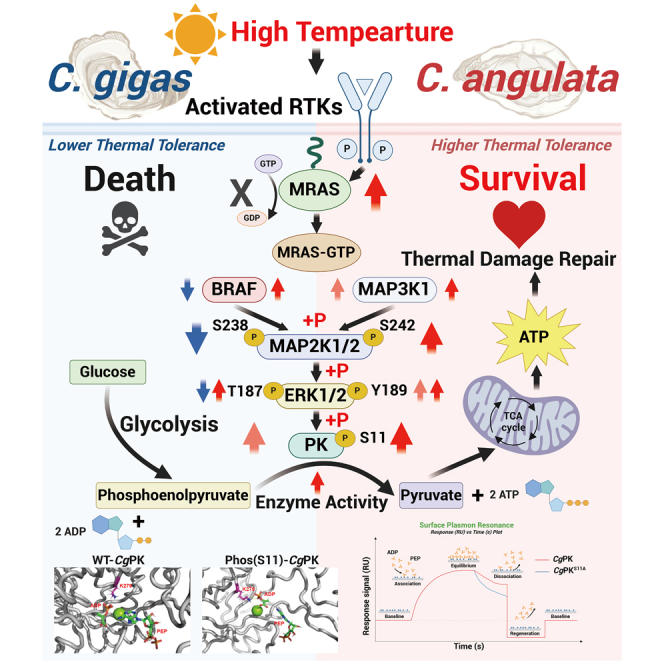

## Introduction

Glycolysis serves as a central pathway in metabolism and plays a vital role in regulating organism’s responses to abiotic stressors, such as temperature.^1^ Previous studies have shown that key genes in glycolysis exhibit differential expression, protein abundance, and enzyme activity in populations or closely related species with divergent temperature tolerance across multiple taxonomic groups,^2^^,^^3^^,^^4^^,^^5^^,^^6^^,^^7^^,^^8^^,^^9^ indicating that the energy metabolism represented by glycolysis is an important stress tolerance effect pathway. While the differential expression of many genes related with glycolysis has provided important insights into long-term biological adaptation to environmental variations, many adaptive responses occur rapidly through post-translational modifications (PTMs) of existing proteins, such as reversible phosphorylation.^10^^,^^11^ Phosphorylation is one of the most extensively studied PTMs, which integrates signal transduction, gene expression, and metabolism in biological stress responses^1^ by influencing conformational changes and localization to regulate the substrate binding capacity of glycolytic enzymes^12^ and control more central metabolism than do changes in protein abundance.^13^^,^^14^ However, despite extensive phosphoproteomics studies revealing diverse phosphorylation dynamics of glycolytic enzymes in response to stress, reliable characterization of the impact of these phosphorylations on enzyme properties has been limited to only a few studies in plants^12^^,^^15^ and model organisms,^16^ with no corresponding research conducted in marine organisms.

Pyruvate kinase (PK) is the key rate-limiting enzyme in the final step of glycolysis, which catalyzes the transfer of a phosphate group from phosphoenolpyruvate (PEP) to ADP, synthesizing ATP and pyruvate, thereby controlling the rate of biological glycolysis and energy production.^17^ In marine organisms, extensive researches have used PK expression or enzyme activity as important indicators to measure stress tolerance and limits.^18^^,^^19^^,^^20^^,^^21^^,^^22^^,^^23^^,^^24^^,^^25^ Furthermore, PK has been reported to be influenced by phosphorylation modifications in human cells. For example, phosphorylation of PKM2 at the Tyr105 site by FGFR1^26^ or CDK6^27^ disrupts the binding of PKM2 cofactor fructose-1,6-bisphosphate, inhibiting the formation of active tetrameric PKM2. Phosphorylation of PKM2 at the Thr454 site by PIM2 promotes glycolysis.^28^ However, there is currently no relevant report regarding PK phosphorylation-mediated activity regulation in marine animals.

Oysters, as representative species in marine mollusks, are distributed worldwide and possess significant economic and ecological values.^29^^,^^30^ Furthermore, oysters inhabit the excessively stressful intertidal zone, which has caused the evolution of perfect tolerance to such conditions,^31^ making them an ideal model organism for revealing the regulatory mechanisms underlying stress adaptation mediated by phosphorylation in marine organism. *Crassostrea gigas* (*C. gigas*) and *Crassostrea angulata* (*C. angulata*) are two allopatric congeneric oyster species that adapt to relatively cold and warm habitats (Northern and Southern China coasts), respectively, exhibiting divergent heat tolerance.^32^^,^^33^^,^^34^^,^^35^^,^^36^ Previous study has found differential protein phosphorylation patterns of PK Ser11 in response to high-temperature stress between *C. gigas* and *C. angulata*.^8^ Specially, the upregulation magnitude of phosphorylation was higher in *C. angulata* compared to *C. gigas*. Therefore, comparative studies between these two species will contribute to unraveling the phosphorylation function of PK (Ser11) in marine organisms and its upstream regulatory cascade pathways, and provide new insights into the role of PTMs, such as phosphorylation, in shaping temperature adaptation, and biological diversity.

In this study, we confirmed the conservation of the PK Ser11 site in bivalves and gastropods through sequence alignment. Through site mutation, molecular dynamics simulations, and surface plasmon resonance (SPR) experiments, we validated that phosphorylation at Ser11 enhances the binding of PK to substrates, thereby increasing enzyme activity. Combining a series of molecular assays, we demonstrated that MAPK/ERK is the upstream kinase and regulatory pathway for this site. Furthermore, the MAPK-PK pathway responded to temperature elevation and exhibited inter-species differentiation in two species during reciprocal transplant experiment, indicating its involvement in temperature adaptation. Our findings elucidate the existence of a unique phosphorylation-mediated energy metabolism signaling transduction cascade in marine mollusks, expanding our understanding of the evolution and function of cross-talk between classical pathways.

## Results

### The PK Ser11 residue is conserved in Bivalvia and Gastropoda

**Figure 1:**
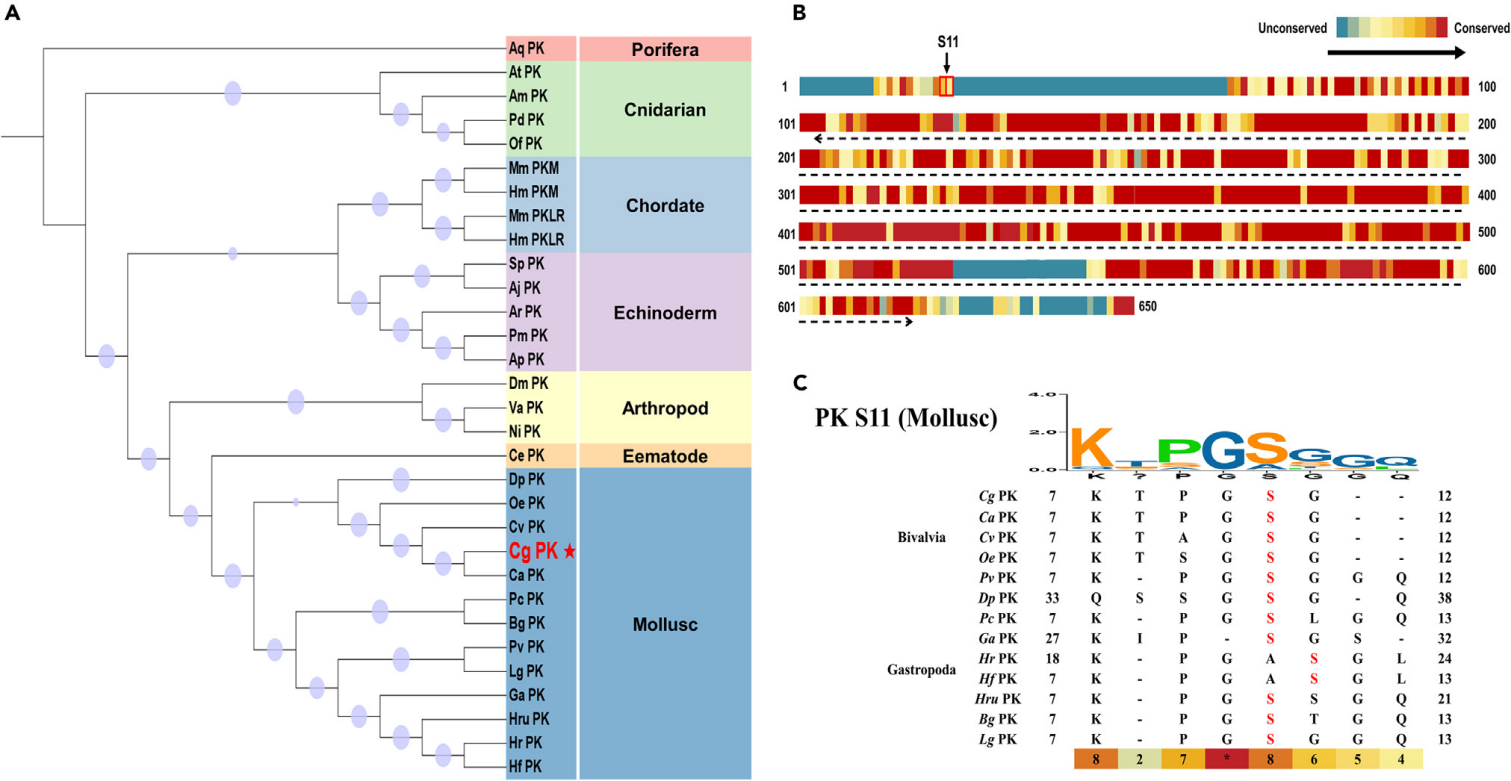
Phylogenetic analysis and sequence alignment of PK genes (A) Phylogenetic tree of PK genes from representative animals across metazoans. Bootstrap support values are indicated by sizes on nodes of phylogenetic tree. The background colors of sequence name represent different phyla. The *Cg*PK genes are marked with red dotted lines. (B) Conservative analysis of PK sequences in metazoan. (C) The sequence alignment surrounding S11 on *Cg*PK.

A total of 29 representative species across different evolutionary positions on the metazoan phylogenetic tree were selected to construct a systematic phylogenetic tree based on PK protein sequences. The results showed that, except for the known vertebrates (human and mouse), the PK genes in the genomes of the remaining species were single-copy genes (Figure 1A). Subsequently, sequence alignment revealed that the Ser11 residue was located in the non-conserved N-terminal domain of the overall sequence (Figure 1B), and it is conserved only in Bivalvia and Gastropoda (Figure 1C).

### The PK Ser11 increases enzyme activity

**Figure 2:**
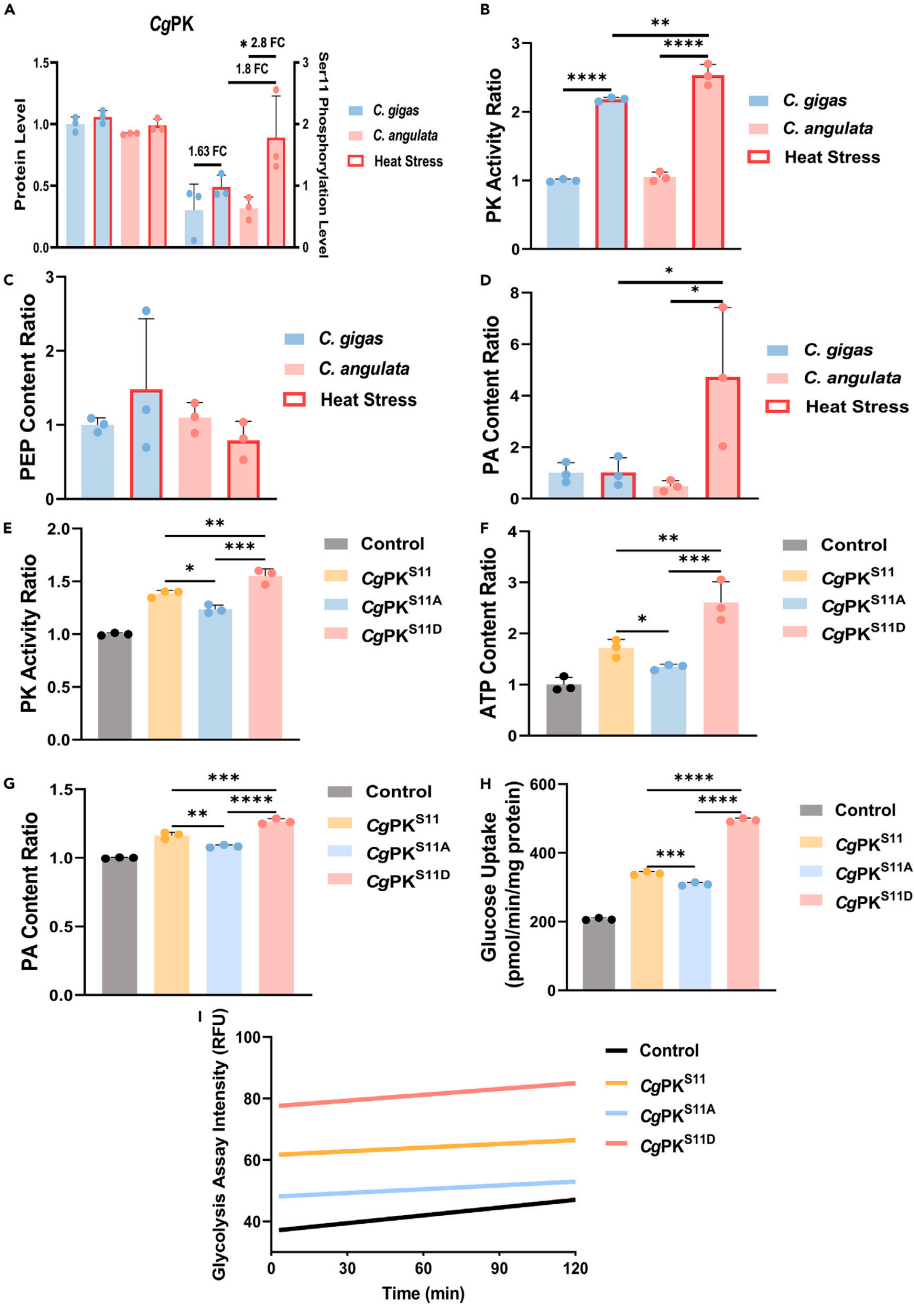
The phosphorylation of conserved Ser11 in *Cg*PK enhances its enzyme activity (A) The phosphorylation levels of Ser11 site of *Cg*PK in gill tissues of *C. gigas* and *C. angulata* during heat stress, which was obtained from our previous study.^8^*In Vivo* PK activity assay (B–I) (*n* = 3; B), PEP content (*n* = 3; C) and PA content (*n* = 3; D) of *C. gigas* and *C. angulata* during heat stress. *In Vivo* PK activity assay (*n* = 3; E), ATP content ratio (*n* = 3; F), PA content ratio (*n* = 3; G), glucose uptake assay (*n* = 3; H) and glycolysis rate assay (*n* = 3; I) of HEK293T cells were transfected with Flag-*CgPk*/Flag-*CgPk*^*S11A*^/Flag-*CgPk*^*S11D*^. All data are presented in the form of mean ± SD. Significant differences among groups were marked with ^∗^*p* < 0.05, ^∗∗^*p* < 0.01, ^∗∗∗^*p* < 0.001, and ^∗∗∗∗^*p* < 0.0001. “ns” indicates non-significant differences.

Previous study has observed that the protein content of PK did not respond to temperature elevation and showed no significant difference between *C. gigas* and *C. angulata*, but the phosphorylation level of the Ser11 residue exhibited divergent heat response pattern.^8^ Specifically, the phosphorylation level of Ser11 in *C. angulata* was significantly upregulated (*p* < 0.05, 2.8-fold), while it showed a slight increase in *C. gigas* (1.63-fold). Additionally, the phosphorylation level of Ser11 in *C. angulata* was higher than that in *C. gigas* after heat stress (1.8-fold; Figure 2A). Further investigation focused on the gill tissue of *C. gigas* and *C. angulata* during heat stress also demonstrated similar differences in PK enzyme activity, PEP content, and PA content. The PK enzyme activity was significantly upregulated in both *C. gigas* and *C. angulata* under heat stress. However, the PK enzyme activity in *C. angulata* was significantly higher than that in *C. gigas* after heat stress (*p* < 0.01, Figure 2B). And the PEP content was lower in *C. angulata* after heat stress compared to *C. gigas* (Figure 2C), while the PA content showed the opposite trend (*p* < 0.05, Figure 2D). Based on single site mutation, we constructed Flag-*CgPk* and its Ser11 mutants (Flag-*CgPk*^*S11A*^ [mimicking dephosphorylation] and Flag-*CgPk*^*S11D*^ [mimicking phosphorylation]). After transfection, the results showed that the phosphorylation of the Ser11 residue significantly enhanced PK enzyme activity (*p* < 0.05; Figure 2E), ATP content (*p* < 0.05; Figure 2F) and PA content (*p* < 0.05; Figure 2G), and significantly improved glucose uptake rate (*p* < 0.01; Figure 2H) and glycolysis rate (*p* < 0.001; Figure 2I).

### The PK Ser11 enhances substrate binding capacity

**Figure 3:**
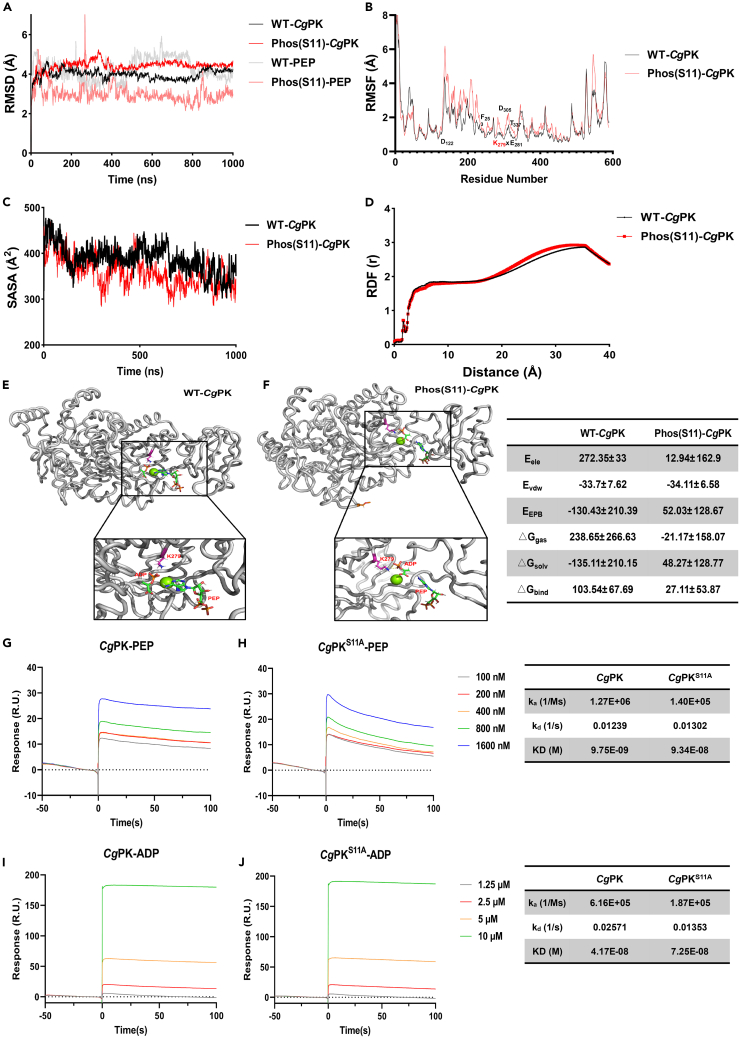
Evaluation of enzyme-substrate binding capacity of PK S11 phosphorylation (A) The time course of RMSD values of the wild type system (black) and the S11-phospho system (red). (B) The time course of RMSF values of the two systems. Those residues with high RMSF values are labeled. (C) The time course of SASA values of the two systems. (D) The RDF values around K279 of PK. The structure of PEP: PK complex for wild type system (E) and the S11-phospho system (F). The interaction between *Cg*PK or *Cg*PK^S11A^ and PEP (G and H) or ADP (I and J) detected by SPR.

We constructed two systems (*Cg*PK-PEP and *Cg*PKPhos S11-PEP) to perform 1,000 ns molecular dynamics simulations. To evaluate the structural stability of the systems, the root-mean-square deviation (RMSD) of the protein backbone C-α atoms was calculated using AmberTools22 software. As shown in Figure 3A, the RMSD values of the wild-type PK protein conformation and the S11 phosphorylated protein conformation showed significant fluctuations during the first 300 ns, with the RMSD values of the S11 phosphorylated protein conformation higher than that of the wild-type PK protein conformation. However, the RMSD value of the Phos(S11)-PEP conformation remained lower than that of the WT-PEP conformation. Root-mean-square fluctuation (RMSF) is an important parameter in protein molecular dynamics simulations that describes the deviation of each atom in a protein from its equilibrium position during the simulation. From Figure 3B, it can be observed that the RMSF values of most residues in the S11 phosphorylated PK system increased to varying degrees, especially at active sites in the PK protein: D122, F253, K279xE281, D305, and T337. The solvent accessible surface area (SASA) refers to the total area on the molecular surface that is accessible to solvent molecules. The Figure 3C demonstrated that the SASA values of the S11 phosphorylated system are lower than that of the wild-type system. The radial distribution function (RDF) describes the distribution of water molecules around a central point in space. The results in Figure 3D showed that the density of water molecules near the K279 core catalytic site in the S11 phosphorylated system is higher than that in the wild-type system.

Non-covalent interactions, such as hydrogen bonds, hydrophobic interactions, and salt bridges, play important roles in the structure formation and biological functions of macromolecules. In the 1,000 ns simulations of the wild-type system and the S11 phosphorylated system, we examined the formation of hydrogen bonds between PEP/ADP and surrounding PK residues, as shown in Table 1. The results showed that the wild-type and S11 phosphorylated systems formed 29 and 50 hydrogen bonds, respectively. And the hydrogen bonds in the S11 phosphorylated system had longer durations compared to those in the wild-type system. It is worth noting that the S11 phosphorylated system exhibited unique hydrogen bonds between PEP and the K279 core catalytic residue, represented as UNK_591@O2-LYS_279@HZ1, UNK_591@O2-LYS_279@HZ2, UNK_591@O3-LYS_279@HZ1, UNK_591@O3-LYS_279@HZ2, UNK_591@O3-LYS_279@HZ3, UNK_591@O4-LYS_279@HZ1, and UNK_591@O4-LYS_279@HZ3, which were completely absent in the wild-type system. Moreover, the calculation of enzyme-substrate binding affinity by MM/GBSA method showed that the binding free energy in the S11 phosphorylated system is lower than that in the wild-type system (ΔG_bind_; Figures 3E and 3F).

**Table 1:** Properties of the formed hydrogen bonds between PEP/ADP and its adjacent PK residues

H-Bond	Wide Type System				S11-Phospho System			
	Occupancy(%)	Distance(Å)	Angle(Å)	Lifetime	Occupancy(%)	Distance(Å)	Angle(Å)	Lifetime
UNK_591@O2-ARG_86@HH22	17.37	2.85	154.91	14.33	10.57	2.89	159.68	5.76
UNK_591@O3-ARG_86@HH12	10.87	2.77	158.08	18	6.37	2.81	161.74	18.04
UNK_591@O5-ASN_88@HD22	29.3	2.89	155.79	34.33	19.37	2.9	156.09	8.9
UNP_592@O6-LYS_124@HZ3	2.07	2.86	156.99	2.33	1.73	2.86	153.42	1.1
UNP_592@O8-LYS_124@HZ2	2.6	2.82	150.85	2.33	8.27	2.78	156.36	1.3
UNP_592@O8-LYS_124@HZ3	2.93	2.71	155.97	3.67	8.8	2.77	154.54	1.33
UNP_592@O9-LYS_124@HZ2	2.3	2.82	152.43	2.67	3.97	2.78	155.99	1.34
UNP_592@O9-LYS_124@HZ3	2.43	2.78	151.64	3	3.67	2.78	157.97	1.58
UNP_592@O10-LYS_124@HZ1	3.93	2.77	161.71	5.67	5.93	2.76	153.76	1.38
UNP_592@O10-LYS_124@HZ2	2.9	2.79	155.55	4	5.57	2.75	156.88	1.41
UNP_592@O8-LYS_215@HZ1	1.57	2.79	159.92	2	1.03	2.79	149.09	1.39
UNK_591@O6-LYS_376@HZ3	0.5	2.7	163.31	1	0.8	2.79	161.97	1.07
UNK_591@O2-ARG_86@HH12	12.57	2.88	154.02	33	–	–	–	–
UNK_591@O3-ARG_86@HH22	15.4	2.88	147.88	12.67	–	–	–	–
UNK_591@O6-ASN_88@HD21	9	2.95	153.31	5.33	–	–	–	–
UNK_591@O6-ASN_88@HD22	1.2	2.95	155.08	6.67	–	–	–	–
UNK_591@O5-SER_90@HG	5.9	2.78	161.28	9.33	–	–	–	–
UNP_592@O6-LYS_124@HZ1	1.63	2.8	152.26	2.67	–	–	–	–
UNP_592@O9-LYS_124@HZ1	2.77	2.81	155.93	2.67	–	–	–	–
UNP_592@O6-ARG_129@HH12	1.07	2.92	152.7	4.33	–	–	–	–
UNP_592@O8-ARG_129@HH22	16.67	2.87	153.42	23	–	–	–	–
UNP_592@O9-ARG_129@HH12	8.77	2.87	152.99	24.67	–	–	–	–
UNP_592@O10-ARG_129@HH22	34.57	2.82	154.44	55	–	–	–	–
UNP_592@O4-LYS_215@HZ2	0.27	2.97	152.92	1	–	–	–	–
UNP_592@O7-LYS_215@HZ1	1.53	2.85	153.66	2	–	–	–	–
UNP_592@O7-LYS_215@HZ3	1.73	2.78	157.17	2.67	–	–	–	–
UNP_592@O9-LYS_215@HZ2	1.5	2.83	162.2	2.33	–	–	–	–
UNP_592@O9-LYS_215@HZ3	1.1	2.76	154.97	1.67	–	–	–	–
UNP_592@O10-LYS_215@HZ2	2.73	2.81	162.17	2.67	–	–	–	–
UNK_591@O1-ASN_88@HD22	–	–	–	–	1.67	2.9	143.8	1.4047
UNK_591@O5-ASN_88@HD21	–	–	–	–	14.07	2.98	155.09	2.0635
UNP_592@O6-LYS_124@HZ2	–	–	–	–	1.1	2.86	150.77	1.1196
UNP_592@O7-LYS_124@HZ1	–	–	–	–	2.13	2.85	153.92	1.1020667
UNP_592@O7-LYS_124@HZ2	–	–	–	–	2.3	2.83	152.27	1.146
UNP_592@O7-LYS_124@HZ3	–	–	–	–	2.03	2.83	154.74	1.125
UNP_592@O8-LYS_124@HZ1	–	–	–	–	7.93	2.76	154.71	1.3321667
UNP_592@O10-LYS_124@HZ3	–	–	–	–	5.47	2.76	154.79	1.2751667
UNP_592@O7-ARG_129@HH21	–	–	–	–	12.33	2.87	154.02	3.5956667
UNP_592@O6-LYS_215@HZ2	–	–	–	–	2.33	2.84	157.95	1.5769333
UNP_592@O8-LYS_215@HZ2	–	–	–	–	1.1	2.8	156.31	1.1938333
UNP_592@O10-LYS_215@HZ1	–	–	–	–	1.33	2.79	155.1	1.0663667
UNP_592@O10-LYS_215@HZ3	–	–	–	–	1.47	2.76	158.94	1.0802
UNP_592@O6-LYS_233@HZ1	–	–	–	–	3.93	2.84	153.46	1.0701667
UNP_592@O6-LYS_233@HZ2	–	–	–	–	3.87	2.91	156.6	1.1028667
UNP_592@O6-LYS_233@HZ3	–	–	–	–	3.97	2.81	156.01	1.1752333
UNP_592@O7-LYS_233@HZ1	–	–	–	–	5.97	2.88	153.59	1.2039333
UNP_592@O7-LYS_233@HZ2	–	–	–	–	6.23	2.84	150.94	1.0967
UNP_592@O7-LYS_233@HZ3	–	–	–	–	6.1	2.84	153.4	1.4685
UNP_592@O8-LYS_233@HZ1	–	–	–	–	3	2.81	154.86	1.3177333
UNP_592@O8-LYS_233@HZ2	–	–	–	–	2.8	2.78	155.85	1.4041333
UNP_592@O8-LYS_233@HZ3	–	–	–	–	3.1	2.83	156.01	1.4102
UNP_592@O9-LYS_233@HZ1	–	–	–	–	1.63	2.79	156.04	1.0984
UNP_592@O9-LYS_233@HZ2	–	–	–	–	1.77	2.84	154.62	1.6135333
UNP_592@O9-LYS_233@HZ3	–	–	–	–	1.53	2.79	154.03	1.0877
UNP_592@O10-LYS_233@HZ1	–	–	–	–	5.47	2.81	154.38	1.2676667
UNP_592@O10-LYS_233@HZ2	–	–	–	–	6.03	2.78	155.43	1.3307333
UNP_592@O10-LYS_233@HZ3	–	–	–	–	5.67	2.81	155.2	1.3040667
UNK_591@O2-LYS_279@HZ1	–	–	–	–	4.17	2.9	150.19	1.1162667
UNK_591@O2-LYS_279@HZ2	–	–	–	–	4.2	2.88	151.45	1.1855
UNK_591@O3-LYS_279@HZ1	–	–	–	–	2.53	2.77	160.87	1.2673
UNK_591@O3-LYS_279@HZ2	–	–	–	–	2.53	2.82	165.29	1.2531
UNK_591@O3-LYS_279@HZ3	–	–	–	–	2.43	2.86	155.52	1.2993333
UNK_591@O4-LYS_279@HZ1	–	–	–	–	3.3	2.83	155.8	1.3571333
UNK_591@O4-LYS_279@HZ3	–	–	–	–	3.37	2.76	156.45	1.1909667
UNK_591@O2-SER_371@HG	–	–	–	–	7.43	2.63	163.67	7.8377333
UNK_591@O3-SER_371@HG	–	–	–	–	6.47	2.66	161.48	13.8869
UNK_591@O4-SER_371@HG	–	–	–	–	10.67	2.63	162.24	8.6064667

Subsequently, the SPR experiments were conducted to validate the results of molecular dynamics simulations and the impact of S11 phosphorylation on enzyme-substrate affinity. Figures 3G and 3H showed the dose-dependent binding of PK and PK^S11A^ proteins with PEP, and the calculated equilibrium dissociation constants (*K*_D_) are 9.75 × 10^−9^ M and 9.34 × 10^−8^ M, respectively. And Figures 3I and 3J showed the dose-dependent binding of PK and PK^S11A^ proteins with ADP, and the calculated equilibrium *K*_D_ are 4.17 × 10^−8^ M and 7.25 × 10^−8^ M, respectively.

### *Cg*ERK1/2 phosphorylates *Cg*PK Ser11

**Figure 4:**
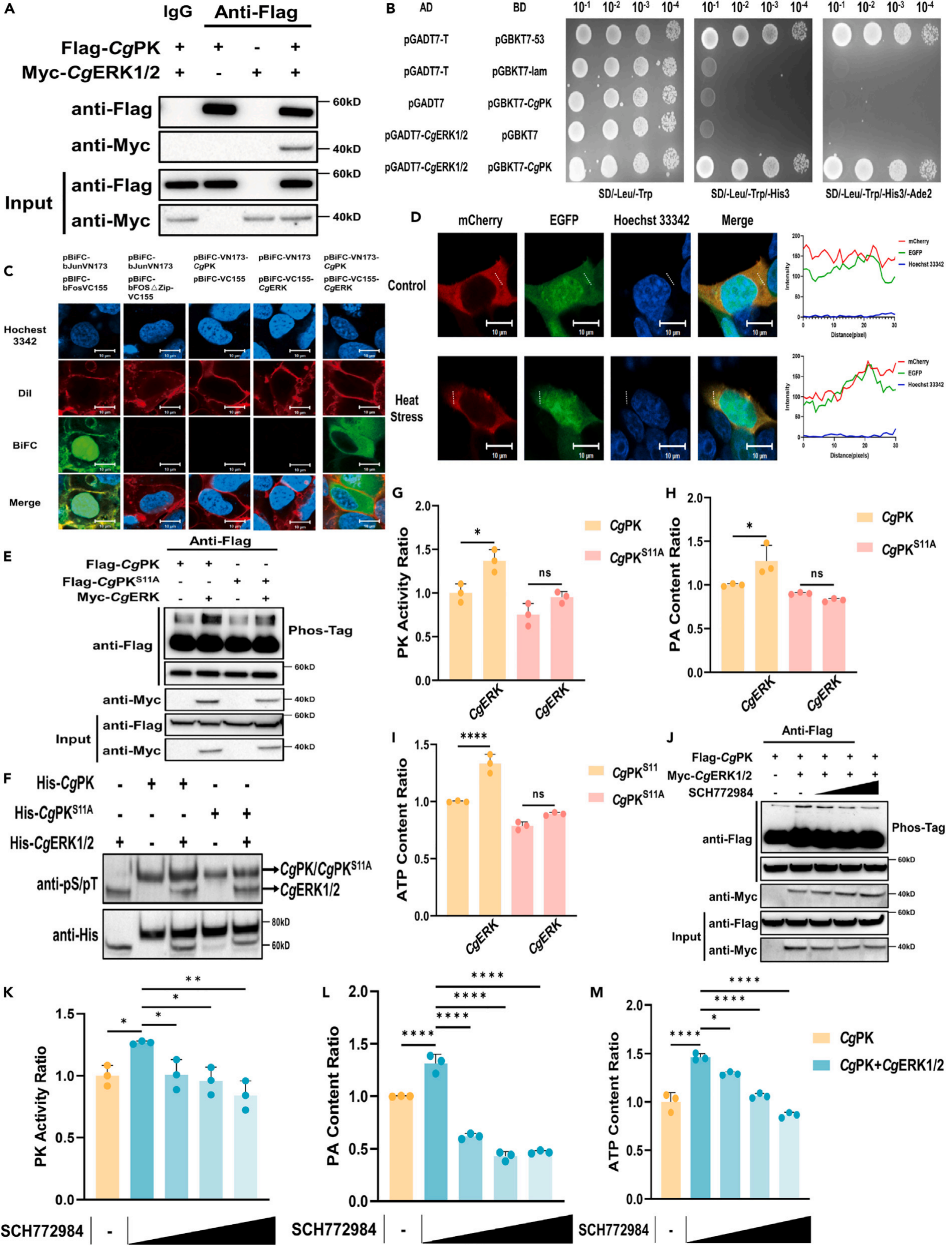
*Cg*ERK1/2 phosphorylates PK Ser11 to enhance its enzymatic activity (A) Co-immunoprecipitation (coIP) of *CgERK1/2* and *CgPK*. (B) Yeast two-hybrid assay between *CgERK1/2* and *CgPK*. (C) BiFC assay of *CgERK1/2* and *CgPK*. (D) Subcellular localization of *CgERK1/2* and *CgPK* in HeLa cells under control and heat stress. (E) *In vivo* phosphorylation assay of *CgERK1/2* on *CgPK* Ser11 site. (F) *In vitro* phosphorylation assay of *CgERK1/2* on *CgPK* Ser11 site. The *in Vivo* PK activity assay (G–M) (*n* = 3; G), PA content (*n* = 3; H) and ATP content (*n* = 3; I) of HEK293T cells transfected with Flag-*CgPk*/Flag-*CgPk*^S11A^ and Myc-*CgErk1/2*. The *In vivo* phosphorylation assay (J), *in Vivo* PK activity assay (*n* = 3; K), PA content ratio (*n* = 3; L) and ATP content ratio (*n* = 3; M) of HEK293T cells transfected with Flag-*CgPk* and Myc-*CgErk1/2* after incubating with ERK inhibitor (SCH772984) for 8 h. All data are presented in the form of mean ± SD. Significant differences among groups were marked with ^∗^*p* < 0.05, ^∗∗^*p* < 0.01, ^∗∗∗^*p* < 0.001, and ^∗∗∗∗^*p* < 0.0001. “ns” indicates non-significant differences.

Based on the results of kinase prediction, the ERK protein was inferred to be the kinase that phosphorylated PK Serr11 (Table S3). Phylogenetic analysis confirmed the identification of oyster ERK protein as ERK1/2 (Figure S1). The co-immunoprecipitation coIP results demonstrated the specific interaction between *Cg*ERK1/2 and *Cg*PK in the lysates of cells co-transfected with Flag-*CgPk* and Myc-*CgErk1/2* (Figure 4A). The yeast two-hybrid (Figure 4B) and BiFC assays (Figure 4C) provided additional evidence supporting the specific interaction between *Cg*ERK1/2 and *Cg*PK, predominantly localized within the cytoplasm. The subcellular localization results indicated that *Cg*ERK1/2, upon heat stress, exhibited nuclear translocation, and fluorescence co-localization analysis revealed their interaction (Figure 4D). The *in vivo* kinase experiment demonstrated that the more pronounced migration bands observed when co-transfected with *Cg*ERK1/2 and *Cg*PK than that with lane 1 that transfected with *Cg*PK alone, and the migration bands of *Cg*PK^S11A^ mutant showed no significant changes regardless of co-transfection with *Cg*ERK1/2 (Figure 4E). Moreover, the *in vitro* kinase experiments also support that co-incubation of *Cg*ERK1/2 with *Cg*PK resulted in a stronger band with anti-pS/pT antibody, which was eliminated upon co-incubation of *Cg*PK^S11A^ and *Cg*ERK1/2 (Figure 4F). Furthermore, *in vivo* co-transfection experiments and measurements of enzyme activity and metabolite contents indicated that *Cg*ERK1/2 can significantly enhance *Cg*PK enzyme activity (*p* < 0.05, Figure 4G), as well as the contents of PA (*p* < 0.05, Figure 4H) and ATP (*p* < 0.0001, Figure 4I), which can be attenuated by PK^S11A^ mutant. The results of cell culture with gradient concentrations of SCH772984 (ERK inhibitor) in combination with co-transfection of *Cg*ERK1/2 and *Cg*PK demonstrated that the inhibition of *Cg*ERK1/2 activity led to a gradient decrease in *Cg*PK phosphorylation levels (Figure 4J), PK enzyme activity (*p* < 0.05, Figure 4K), PA content (*p* < 0.0001, Figure 4L), and ATP content (*p* < 0.05, Figure 4M).

### Divergent phosphorylation of *Cg*ERK1/2^T187 Y189^ by MAPK signaling pathway mediates differential PK Ser11 phosphorylation

**Figure 5:**
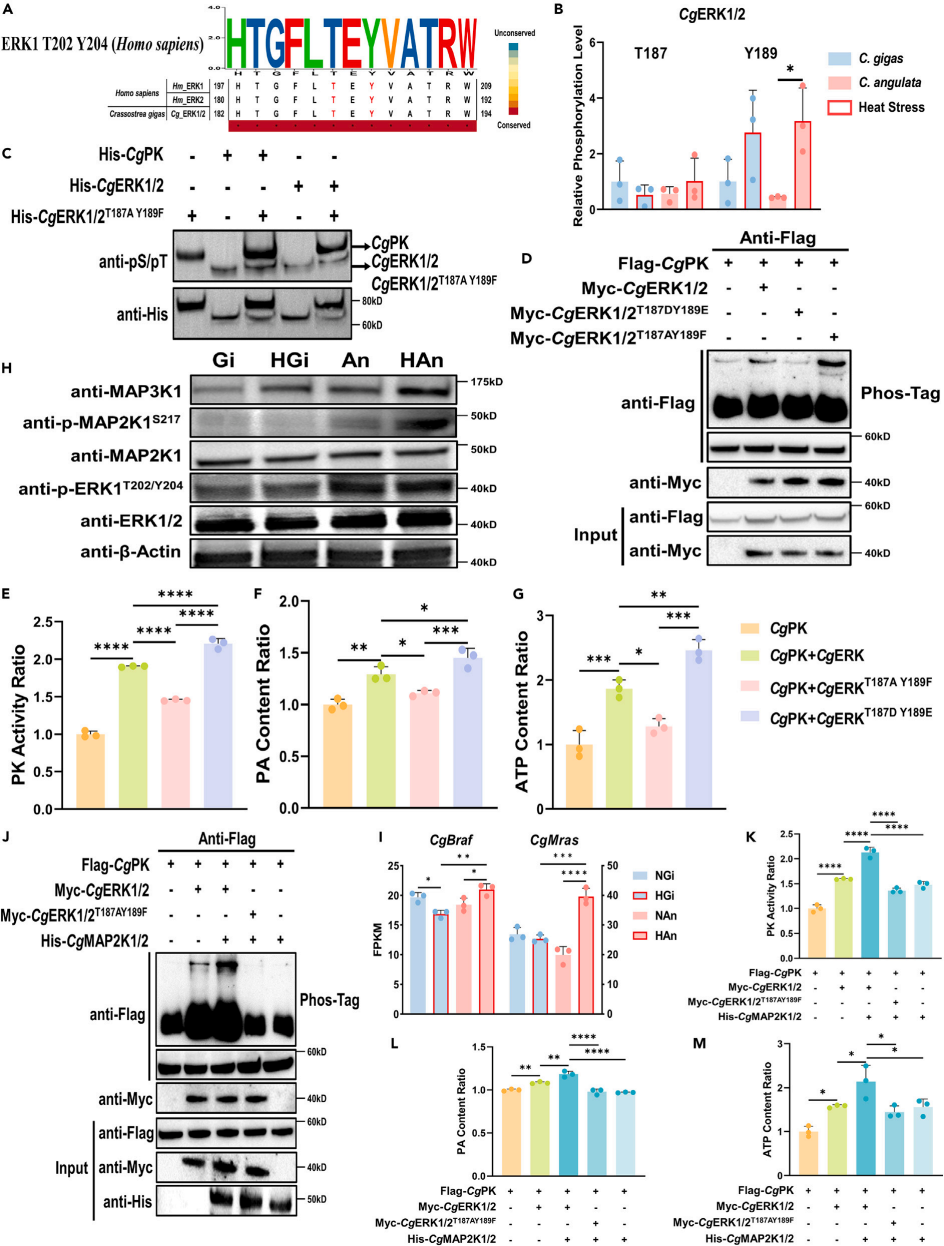
The MAPK/ERK signaling pathway regulates the phosphorylation level of *Cg*PK Ser11 site (A) The sequence alignment of human ERK1, ERK2, and oyster ERK1/2. (B) The phosphorylation levels of Thr187 and Tyr189 sites of *Cg*ERK1/2 in gill tissues of *C. gigas* and *C. angulata* during heat stress, which was obtained from our previous study.^8^ (C) *In vitro* kinase activity assay of *Cg*ERK1/2/CgERK1/2^T187AY189F^ on *Cg*PK Ser11 site. (D) *In vivo* phosphorylation assay of different *Cg*ERK1/2 mutants on *Cg*PK Ser11 site. The *in Vivo* PK activity assay (E–G) (*n* = 3; E), PA content ratio (*n* = 3; F) and ATP content ratio (*n* = 3; G) of HEK293T cells transfected with Flag-*CgPk* and Myc-*CgErk1/2*/Myc-*CgErk1/2*^*T187AY189F*^/Myc-*CgErk1/2*^*T187DY189E*^. (H) The western blotting of proteins extracted from gill tissues of *C. gigas* and *C. angulata* during heat stress with MAPK pathway’s antibodies. Gi and An represent the *C. gigas* and *C. angulata* under control condition. HGi and HAn represent the *C. gigas* and *C. angulata* under heat stress. (I) The expressions of *CgBraf* and *CgMras* from transcriptomic data of *C. gigas* and *C. angulata* during heat stress (*n* = 3).^37^ The *in vivo* phosphorylation assay (J), *in vivo* PK activity assay (K–M) (*n* = 3; K), PA content ratio (*n* = 3; L) and ATP content ratio (*n* = 3; M) of HEK293T cells transfected with Flag-*CgPk*, Myc-*CgErk1/2*/Myc-*CgErk1/2*^*T187AY189F*^*,* and His-*CgMap2k1/2*. All data are presented in the form of mean ± SD. Significant differences among groups were marked with ^∗^*p* < 0.05, ^∗∗^*p* < 0.01, ^∗∗∗^*p* < 0.001, and ^∗∗∗∗^*p* < 0.0001. “ns” indicates non-significant differences.

The sequence alignment results showed that the oyster ERK1/2 residues T187 and Y189 correspond to the conserved active sites T202 and Y204, respectively, in human ERK1 (Figure 5A). Previous phosphoproteomic data from *C. gigas* and *C. angulata* during heat stress revealed divergent patterns of phosphorylation at the *Cg*ERK1/2 T187 and Y189 sites. The phosphorylation level at the *Cg*ERK1/2 T187 site increased in *C. angulata* and decreased in *C. gigas*. And the phosphorylation level at the *Cg*ERK1/2 Y189 increased in two species, but the *C. angulata* possessed stronger upregulation magnitude and significantly higher than that in *C. gigas* (Figure 5B). *In vivo* (Figure 5D) and *in vitro* (Figure 5C) kinase assays, coupled with *Cg*ERK1/2^T187D Y189E^ (mimicking phosphorylation) and *Cg*ERK1/2^T187A Y189F^ (mimicking dephosphorylation) mutants provided compelling evidence that phosphorylation at *Cg*ERK1/2 T187 and Y189 significantly enhanced its kinase activity, causing an elevated phosphorylation level of *Cg*PK Ser11 site. Similarly, the *Cg*ERK1/2 T187 and Y189 phosphorylation mutants significantly increased PK enzyme activity (*p* < 0.0001, Figure 4E), PA content (*p* < 0.05, Figure 4F), and ATP content (*p* < 0.05, Figure 4G).

Western blotting of heat-stressed *C. gigas* and *C. angulata* demonstrated that *Cg*ERK1/2 phosphorylation levels were higher in *C. angulata* than that in *C. gigas* (Figure 5H). The upstream regulators of ERK, including the phosphorylation level of MAP2K1/2 (Figure 5H), expression of *Braf* (*p* < 0.01; Figure 5I) and *Mras* (*p* < 0.001; Figure 5I), and protein content of MAP3K1 (Figure 5H) were significantly higher in HAn (*C. angulata* after heat stress) than in HGi (*C. gigas* after heat stress). The *in vivo* kinase experiment (Figure 5J), PK enzyme activity (*p* < 0.0001; Figure 5L), PA content (*p* < 0.01; Figure 5K), and ATP content (*p* < 0.05; Figure 5M) proved that *Cg*MAP2K1/2 can increase the phosphorylation level of *Cg*PK phosphorylating *Cg*ERK1/2, and significantly improve PK enzyme activity, PA and ATP contents.

### Oyster’s MAPK/ERK/PK regulatory axis exhibits environmental responsiveness

**Figure 6:**
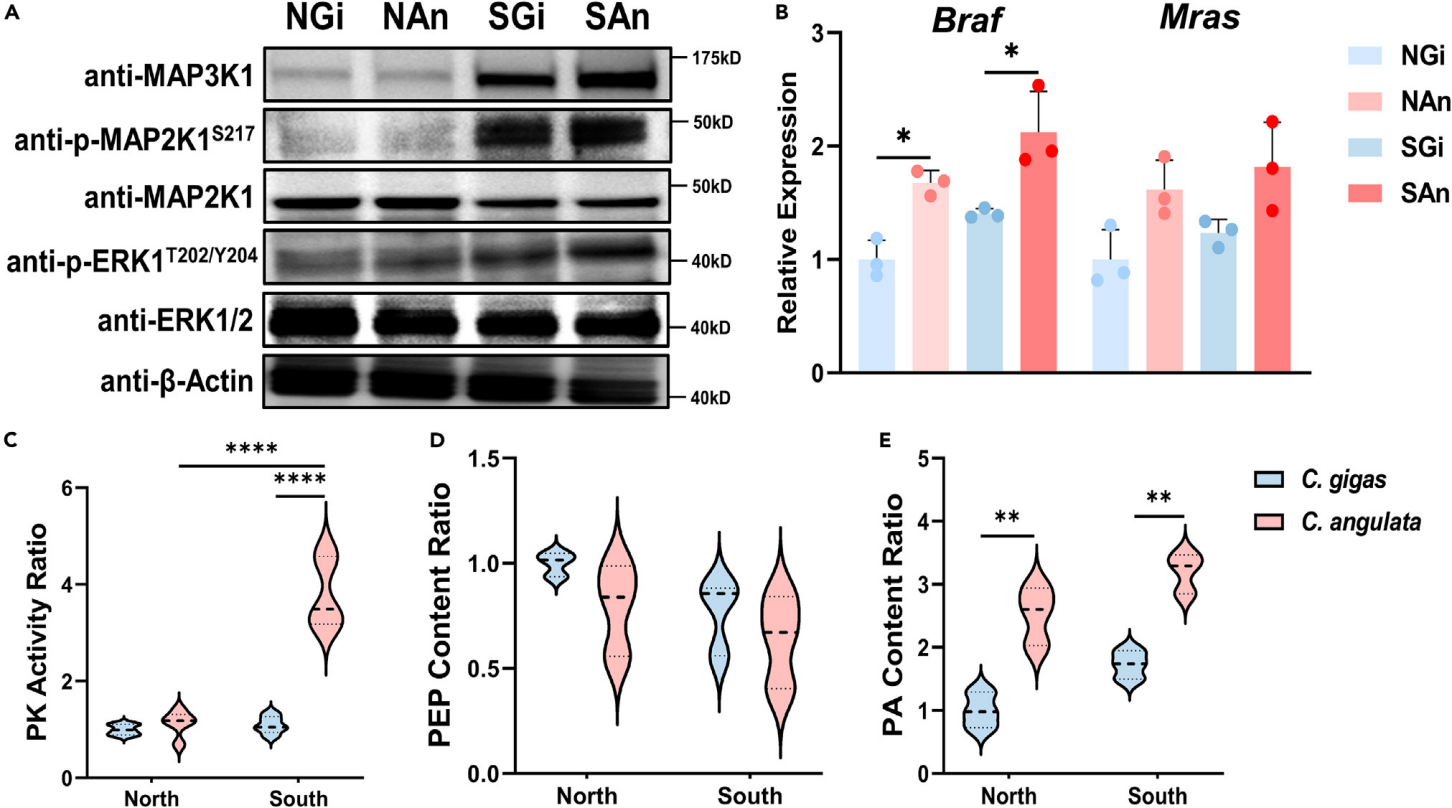
The MAPK/ERK/PK axis demonstrates environmental responsiveness (A) The western blotting of proteins extracted from gill tissues of *C. gigas* and *C. angulata* reared at northern (Qingdao, 35°44′ N) and southern (Xiamen, 24°33′ N) sampling sites. NGi and NAn represent the *C. gigas* and *C. angulata* were reared at northern site. SGi and SAn represent the *C. gigas* and *C. angulata* were reared at southern site. (B) The relative expression of *CgBraf* and *CgMras* of *C. gigas* and *C. angulata* reared at northern (Qingdao, 35°44′ N) and southern (Xiamen, 24°33′ N) sampling sites, which was obtained from our previous study and reanalyzed in this study.^38^ The PK activity assay (C–E) (*n* = 3; C), PEP content ratio (*n* = 3; D) and PA content ratio (*n* = 3; E) of *C. gigas* and *C. angulata* reared at northern (Qingdao, 35°44′ N) and southern (Xiamen, 24°33′ N) sampling sites. All data are presented in the form of mean ± SD. Significant differences among groups were marked with ^∗^*p* < 0.05, ^∗∗^*p* < 0.01, ^∗∗∗^*p* < 0.001, and ^∗∗∗∗^*p* < 0.0001. “ns” indicates non-significant differences.

The 8-month-old F_1_ progeny of *C. gigas* and *C. angulata* were transplanted into their native and nonnative habitats, the northern (Qingdao, 35°44′ N) and southern (Xiamen, 24°33′ N) regions of China, and their corresponding protein phosphorylation and gene expression levels were evaluated after a 3-month reciprocal transplantation between local and non-local environments. Our previous study has showed that the average seawater temperature in the southern habitat (19.64°C) was significantly higher than that in the northern habitat (11.24°C; unpublished data), reflecting the distinct environmental temperature variations between the natural habitats of the two species. Western blotting of reciprocally transplanted *C. gigas* and *C. angulata* demonstrated that the protein levels of *Cg*ERK1/2 and *Cg*MAP2K1/2 remained unaffected by the environment. However, their phosphorylation levels (*Cg*ERK1/2^T187 Y189^ (corresponding to *Hm*ERK1^T202 Y204^) and *Cg*MAP2K1/2^S238^ (corresponding to *Hm*MAP2K1^S218^)) showed a divergent pattern, with higher levels observed in the southern habitat than the northern habitat and in *C. angulata* than in *C. gigas* (Figure 6A). Moreover, the protein content of *Cg*MAP3K1 (Figure 6A), gene expression levels of *CgBraf* and *CgMras* (Figure 6B), the PK enzyme activity (Figure 6C), PEP (Figure 6D), and PA (Figure 6E) contents also demonstrated a similar trend.

## Discussion

In glycolysis, two major regulatory steps are the phosphorylation of fructose-6-phosphate (F6P) by phosphofructokinase to form fructose-1,6-bisphosphate (FBP), and the dephosphorylation of PEP by PK to form pyruvate. Previous studies have demonstrated that the metabolic regulation of PEP is a central hub for both aerobic and anaerobic metabolism.^39^^,^^40^^,^^41^ PK, as a key enzyme controlling this balance, participates in aerobic and anaerobic metabolism under various stressors. On one hand, it promotes glycolysis and the TCA cycle to enhance energy production in aerobic metabolism. On the other hand, it regulates the transition from aerobic to anaerobic metabolism to compensate for insufficient aerobic metabolic capacity. Therefore, PK serves as an important indicator to measure the tolerance and sensitivity of marine organisms in facing environmental stress.^34^^,^^42^^,^^43^ This study discovered that phosphorylation of the conserved PK Ser11 site in mollusks (Bivalvia and Gastropoda) significantly enhances its enzyme activity. Previous study has shown divergent phosphorylation patterns of PK Ser11 in two congeneric oyster species (*C. gigas* and *C. angulata*) under heat stress, which inhabiting different temperature environments.^8^^,^^32^^,^^33^^,^^34^ Specifically, Ser11 phosphorylation is induced by high temperature, and the relatively thermotolerant *C. angulata* exhibits higher upregulation magnitude. The measurements of PK enzyme activity, PEP, and PA contents of *C. gigas* and *C. angulata* confirmed that the *C. angulata* exhibited stronger PK catalytic activity and energy metabolism capacity under heat stress. Furthermore, physiological and biochemical measurements combined with Ser11 site mutation demonstrated that Ser11 phosphorylation can significantly enhance PK enzyme activity, which then increase the glycolysis rate to improve ATP production. Therefore, we suggest that the phosphorylation of PK can rapidly activate the *C. angulata* energy metabolism to mediate differential heat tolerance.

The molecular dynamics simulation results revealed that Ser11 phosphorylation significantly affects the protein conformation of PK and its substrates (PEP and ADP). Among these, the RMSD values of the wild-type PK system were consistently lower than those of the S11 phosphorylated system, indicating that S11 phosphorylation leads to a dynamic and unstable state of the PK protein. While the RMSD values of PEP in the S11 phosphorylated system were consistently lower than those of the wild-type system, indicating that S11 phosphorylation enhances the binding between PK and PEP, making their interaction in a more stable bound state. In the S11 phosphorylated system, higher RMSF values were observed for most residues, indicating that S11 phosphorylation can enhance the flexibility of PK protein residues (including D122, F253, K279xE281, D305, and T337 etc.), resulting in more prone to undergo conformational changes. It is proved that the HmPKM2 reaction occurs in two steps. The first step involves the transfer of a phosphate group from PEP to ADP, resulting in the formation of an enol intermediate and ATP. The second step involves the protonation of the enol intermediate, leading to the formation of pyruvate.^44^ The D122 (corresponding to HmPKM2 D122) coordinates with the K^+^ atom through its side-chain oxygen atom, thereby dissipating the developing negative charge during the phosphoryl transfer reaction.^45^ And the K279 is conserved in all human PK isozymes and facilitates the phosphoryl transfer step by stabilizing the pentavalent phosphate transition state.^46^ Additionally, the E281 (corresponding to HmPKM2 E272) and D305 (corresponding to HmPKM2 D296) coordinates with the Mg2^+^ ion, mediating the interaction between the Mg2^+^ atom and the enol intermediate, thereby influencing the closure of the substrate pocket.^45^ T337 (corresponding to HmPKM2 T328) plays a crucial role in the formation of the hydrogen-bonding network and activation of water molecules, thus playing an important role in the protonation of the enol pyruvate’s ethylene carbon atom.^47^ Further hydrogen bond analysis revealed that the PK protein formed more hydrogen bonds with substrates in the S11 phosphorylation system, indicating that S11 phosphorylation enhanced the interaction between PK and PEP/ADP. It is noteworthy that there are seven specific hydrogen bonds between PEP and the core catalytic residue K279 in the S11 phosphorylation system, implying that S11 phosphorylation plays a crucial role in stabilizing the 591@O-K279@HZ interaction. The results of molecular dynamics simulations were further validated by SPR experiments, which demonstrated that S11 phosphorylation significantly reduced the energy barriers between PK protein and PEP/ATP, thereby enhancing the protein-substrate affinity and improving enzymatic activity.

Based on kinase prediction and subsequent functional validation, we confirmed that the MAPK/*Cg*ERK1/2 kinase phosphorylated Ser11 site of *Cg*PK, then enhanced the glycolysis pathway to resist heat stress. ERK1 and ERK2 are highly conserved effector kinases within the MAPK family, which have been reported that phosphorylate numerous substrates and regulating various conserved biological processes.^48^ Yang et al. has discovered that ERK1/2 can phosphorylate the Ser74 and Ser37 sites of PKM2, leading to its interaction with PIN1 and subsequent conformational changes that convert it from an active tetramer to a monomer. The monomeric form of PKM2 can further translocate to the nucleus and act as a histone kinase, upregulating the expression of c-Myc and cyclin D1, ultimately promoting the Warburg effect (cancer cells preferentially utilize glycolysis to produce energy even in aerobic environments, thereby promoting tumor growth and proliferation) and cell cycle progression, respectively.^49^^,^^50^ Additionally, PIM2 has been reported to phosphorylate the Thr454 site of PKM2, enhancing its protein abundance and glycolytic capacity.^28^ Indeed, current researches on PK phosphorylation primarily focus on the phosphorylation regulation of its major isoform, PKM2, which is involved in the regulation of the Warburg effect and cancer progression.^26^^,^^27^^,^^28^^,^^51^ Therefore, this study firstly reports a conserved non-biological stress-induced phosphorylation site (S11) in marine organisms, which, upon phosphorylation by ERK1/2 kinase, enhances its enzyme activity and increases glycolytic rate to promote ATP production in response to heat stress.

Further measurements of phosphorylation and expression levels in key kinases and regulatory factors of the classical MAPK pathway showed that *C. angulata* demonstrated a stronger activation pattern in classical MAPK pathway than *C. gigas* during heat stress and under increased wild environmental temperature, as evidenced by higher phosphorylation levels of *Cg*ERK1/2 at T187 and Y189 and *Cg*MAP2K1/2 at S238 (corresponding to *Hm*MAP2K1 S217), the protein content of MAP3K1, and the expression levels of *CgBraf* and *CgMras*. It has been reported that the MAPK/ERK pathway is involved in the heat stress response of marine invertebrates.^52^^,^^53^^,^^54^^,^^55^^,^^56^ This pathway induces the expression of heat shock proteins,^57^^,^^58^ alleviates oxidative stress and mitochondrial damage,^59^^,^^60^ and ultimately promotes cell survival and inhibits apoptosis.^61^ The results of site mutations of *Cg*ERK1/2 and co-transfection with *Cg*MAP2K1/2 proved that *Cg*MAP2K1/2 can enhance PK enzyme activity by increasing the phosphorylation levels of T187 and Y189 in *Cg*ERK1/2. The classical MAPK pathway showed that MAP2K1/MAP2K2 activates ERK1/ERK2 through phosphorylation its Thr-202/185 and Tyr-204/187 sites.^62^ And the Ser-218/222 and Ser-222/226 sites of MAP2K1/MAP2K2 are phosphorylated by RAF or MAP3K1,^63^ and RAF is recruited to the membrane by Ras-GTP and activated by other kinases such as PKA, PAK, and SRC.^64^ In this study, MRAS exhibited thermo-responsiveness, which has been reported that counteracts inhibitory phosphorylation events on the RAF protein family through the SHOC2-MRAS-PP1C complex to enhance MAPK signal transduction.^65^ Furthermore, there is evidence that during heat stress, RAS can be activated by receptor tyrosine kinases (RTKs) to regulate cell proliferation and survival through downstream MAPK cascades, similar to cell proliferation and survival induced by growth factor stimulation.^66^ Our previous study indicated that under heat stress, there are significant differences in the protein abundance and phosphorylation levels of RTKs, such as FGFR3, *C. gigas* and *C. angulata*.^8^ Therefore, our study suggests the existence of a high-temperature RTK-MRAS-BRAF-MAPK-PK signaling cascade in mollusks, with the core components being the activation of MAPK/ERK and phosphorylation of its substrate PK (Ser11). This cascade responds to increased environmental temperature and exhibits interspecific divergence, indicating its potential involvement in regulating temperature adaptation and differentiation in organisms.

In conclusion, this study uncovers a unique high-temperature-induced crosstalk mechanism between the MAPK and glycolysis pathways in mollusks. Figure 6F provides a schematic representation of the high-temperature RTK-MRAS-BRAF-MAPK-PK signaling cascade in oysters during heat stress. Our results firstly reported the presence of a heat-induced conserved phosphorylation site (S11) in the key rate-limiting enzyme of glycolysis, PK, in mollusks (Bivalvia and Gastropoda). The phosphorylation at this site enhanced substrate binding and increases PK enzyme activity, thereby promoting ATP production to aid in heat-induced damage repair. Further validation of upstream kinases and regulatory pathways revealed that the PK Ser11 site was phosphorylated by ERK1/2 kinase and regulated by the classical MAPK pathway. The differential heat response and temperature adaptation patterns observed in the high temperature-RTK-MRAS-BRAF-MAPK-PK signaling cascade between two closely related but ecologically divergent species, *C. gigas* (relatively thermosensitive) and *C. angulata* (relatively thermotolerant), suggests its involvement in shaping their temperature adaptation divergence. This study highlights the presence of complex and unique phosphorylation-mediated stress response regulatory network in the conserved glycolysis pathway of marine organisms, which suggests that PTMs play a crucial role in regulating energy metabolism pathways to mediate temperature adaptation. Furthermore, it provides new insights into the evolution and function of the crosstalk mechanisms between existing classical pathways.

### Limitations of the study

Due to the lack of immortal cell lines in mollusks such as oysters, many of the results in this study were obtained using the human model cell line HEK293T. Therefore, there may be some deviations between the results and the *in situ* situations in oysters. Additionally, marine mollusks currently lack large-scale and mature genetic manipulation techniques, which prevent us from performing the substitution of the PK Ser11 site at the individual oyster. Another limitation of this study is that researches on PTMs such as phosphorylation in marine organisms are still in infancy stage, thus limited omics data hinders our understanding of whether phosphorylation of the Ser11 site in PK responds to other biotic and abiotic stressors.

## STAR★Methods

### Key resources table

**Table:** 

REAGENT or RESOURCE	SOURCE	IDENTIFIER
**Antibodies**		

Mouse monoclonal to Flag-tag	ZENBIO	Cat#390002
Mouse monoclonal to Myc-tag	ZENBIO	Cat#390003
Mouse monoclonal to beta Actin	ZENBIO	Cat#200068-8F10
Mouse monoclonal to His-tag	ZENBIO	Cat#230001
Rabbit monoclonal to ERK1/ERK2	ABclonal	Cat#A16686; RRID: AB_2770274
Rabbit monoclonal to Phospho-ERK1-T202/Y204 + ERK2-T185/Y187	ABclonal	Cat#AP0472; RRID: AB_2756833
Rabbit monoclonal to MAP2K1/MAP2K2	ABclonal	Cat#A24394
Rabbit monoclonal to Phospho-MAP2K1-S217/MAP2K2-S221	ABclonal	Cat#AP0209; RRID: AB_2771278
Rabbit monoclonal to MAP3K1	ABclonal	Cat#A21490
Phosphoserine/threonine Antibody	ECM Biosciences	Cat#PP2551; RRID: AB_1184778
HRP-labeled Goat Anti-Mouse/Rabbit IgG(H+L)	Epizyme	Cat#LF101; RRID: AB_3083706
HRP-labeled Goat Anti-Mouse/Rabbit IgG(H+L)	Epizyme	Cat#LF102; RRID: AB_3083707

**Bacterial and virus strains**		

*E. coli* Trelief 5α	Tsingke Biotechnology	Cat#TSC01
E. coli Transetta (DE3)	Tsingke Biotechnology	Cat#TSC-E01

**Chemicals, peptides, and recombinant proteins**		

Pyruvate kinase assay kit	COMIN	Cat#PK-1-Y
Pyruvate (PA) assay kit	COMIN	Cat#PA-1-Y
ATP content assay kit	COMIN	Cat#ATP-1-Y
Glucose uptake assay (Fluorometric, Direct Glucose)	Abcam	Car#ab234043
Glycolysis Assay [Extracellular acidification]	Abcam	Cat#ab197244
CgPK	This paper	N/A
CgPK^S11A^	This paper	N/A
Ni-NTA agarose	Qiagen	Cat#30230
Series S Sensor Chip CM5	GE Healthcare	Cat#29104988
HBS-EP+ buffer	GE Healthcare	Cat#BR100669
Amine Coupling Kit	GE Healthcare	Cat#BR100050
PBS-P+ buffer	GE Healthcare	Cat#28995084
Phosphoenolpyruvic acid potassium	MCE	Cat#HY-W008807
Adenosine 5'-diphosphate	MCE	Cat#HY-W010918
Glycine 1.5	GE Healthcare	Cat#BR100354
Flag-tag Protein IP Assay Kit with Magnetic Beads	Beyotime Biotechnology	Cat#P2181S
GAL4 Yeast Two-Hybrid Media Kit	Coolaber	Cat#YM2001-1
Lipofectamine 3000	Invitrogen	Cat#L3000075
RPMI Medium 1640	Biological Industries	Cat#01-100-1A
Fetal bovine serum	Biological Industries	Cat#04-121-1A
DMEM	Biological Industries	Cat#01-052-1A
Cell lysis buffer for Western and IP	Beyotime Biotechnology	Cat#P0013
Protease inhibitor cocktail	Beyotime Biotechnology	Cat#P1005
Phos-tag™ Acrylamide	FUJIFILM Wako Pure Chemical Corporation	Cat#304-93526
Omni-Easy™ One-step Color PAGE Gel Rapid Preparation Kit	Epizyme	Cat#PG210
SCH772984	MCE	Cat#HY-50846

**Experimental models: Cell lines**		

HEK293T	Procell Life Science & Technology	Cat#CL-0005
HeLa	Procell Life Science & Technology	Cat#CL-0101

**Experimental models: Organisms/strains**		

*Crassostrea gigas*	A local farm in Qingdao, Shandong Province	N/A
*Crassostrea angulata*	A local farm in Xiamen, Fujian Province	N/A

**Recombinant DNA**		

pCMV-Flag-CgPK variants (full length and variations)	This paper	N/A
pET32a-CgPK variants (full length and variations)	This paper	N/A
pCMV-Mya-CgERK1/2 (full length and variations)	This paper	N/A
pET32a-CgERK1/2 variants (full length and variations)	This paper	N/A
pGADT7-CgERK1/2	This paper	N/A
pGBKT7-CgPK	This paper	N/A
pBiFC-VN173-CgPK	This paper	N/A
pBiFC-VC155-CgERK1/2	This paper	N/A
pCMV-mCherry-CgPK	This paper	N/A
pCMV-EGFP-CgERK1/2	This paper	N/A
pCMV-His-CgMAP2K1/2		

**Software and algorithms**		

PhyloSuite 1.2.2	Zhang et al.^67^	http://phylosuite.jushengwu.com/dongzhang0725.github.io/
Amber 22	Case et al.^68^	https://ambermd.org/tutorials/
BIAcore	BIAcore Software	https://www.cytivalifesciences.com/en/us/support/software/biacore-downloads#
iGPS1.0	Song et al.^69^	http://igps.biocuckoo.org/
PRALINE	PRALINE Software	http://www.ibi.vu.nl/programs/pralinewww/
Graphpad Prism	GraphPad Software	http://www.graphpad.com/

### Resource availability

#### Lead contact

Further information and requests for resources should be directed to the lead contact, Li Li (lili@qdio.ac.cn).

#### Materials availability

All materials generated in this study are available from the lead contact with a completed Materials Transfer Agreement.

#### Data and code availability

All data reported in this paper will be shared by the lead contact upon request.
• This paper does not report original code.
• Any additional information required to reanalyze the data reported in this paper is available from the lead contact upon request.

### Experimental model and study participant details

#### Animals

Wild adult oyster of *C. gigas* and *C. angulata* were collected from their natural habitat in Qingdao (35°44′ N) and Xiamen (24°33′ N), respectively, and used as broodstock.^36^ The one-generation common garden experiment was conducted to alleviate the environmental effects.^70^ Artificial breeding program including broodstock conditioning, fertilization, and larval cultures, all of which were conducted in hatchery with 22-26°C and 31 ± 1 ‰ seawater. Briefly, the eggs of 30 mature females were mixed and divided into 30 beakers for each species, each fertilized with sperm from one of the 30 males, to maximize parental contribution. Juvenile F_1_ progeny (8 months old) of each species were separated into two groups: one group was deployed at the northern site (35°44′N, Qingdao, Shandong province, China) and the other group was deployed at the southern site (24°33′N, Xiamen, Fujian Province, China). Three wo months later, 10-month-old oysters were sampled from each site or collected to laboratory for subsequent studies.

#### Cell line and bacteria

The cell line HEK293T and HeLa (Procell Life Science & Technology, China) were grown in high-glucose DMEM Medium and RPMI Medium 1640 (Biological Industries, Israel) with 10% fetal bovine serum (Biological Industries, Israel) at 37°C in a 5 % CO_2_ incubator. *E. coli* Trelief 5α and *E. coli* Transetta (DE3) (Tsingke Biological Technology, Beijing, China) strains were cultured in Luria-Bertani (LB) broth supplemented with 100 mg/mL ampicillin or 50 mg/mL kanamycin at 37°C.

### Method details

#### Phylogenetic analysis

The sequence of each PK gene from different species and ERK from human was retrieved from the National Center for Biotechnology Information (NCBI) database. The maximum likelihood phylogenetic tree was reconstructed using PhyloSuite 1.2.2 software.^67^ Sequences alignments were conducted using PRALINE software (http://www.ibi.vu.nl/programs/pralinewww/), and poorly aligned sequences and gaps were removed using Gblocks 9.1b (http://www.phylogeny.fr/one_task.cgi?task_type=gblocks). The optimal models were tested by ModelFinder,^71^ and the WAG+I+G4 (PK) and LG+I+G4 (ERK) model were selected for multi-species tree reconstruction. IQ-TREE integrated into PhyloSuite was used for maximum likelihood tree construction with 1000 bootstrapping replicates.^72^ The cDNA sequences of CgPK, CgERK1/2 and CgMAP2K1/2 were deposited in the GenBank with accession number PP828782, PP828783 and PP828784. See Table S1 for details.

#### Oyster tissue PK enzyme activity and metabolite detection

Gill tissues of *C. gigas* and *C. angulata* from heat stress treatment (37°C (sublethal temperature) for 12 hours) and reciprocal transplant experiment were used for PK enzyme activity and related metabolites measurements. PK enzyme activity was assessed using the Pyruvate kinase assay kit (COMIN, China) following to the manufacturer’s instruction. The LC-MS/MS analysis was performed by Shanghai Bioprofile Technology Co., Ltd (China) to detect the content of phosphoenolpyruvate (PEP) and pyruvate (PA) using Nexera X2 LC-30AD (Shimadzu, Japan) and QTRAP 5500 (AB SCIEX, France).

#### *In vivo* PK Ser11 phosphorylation functional experiments

The full-length CDSs of *CgPk*, *CgPk*^*S11A*^ (mimicking dephosphorylation) and *CgPk*^*S11D*^ (mimicking phosphorylation) were amplified and inserted into pCMV-N-Flag plasmids (Table S2). The Lipofectamine 3000 (Invitrogen, USA) was used to transfect these plasmids into HEK293T cells (Procell Life Science & Technology, China). After 36 hours, the PK enzyme activity assay was performed following the aforementioned procedure. The contents of pyruvate and ATP were measured using pyruvate (PA) assay kit and ATP content assay kit (COMIN, China) following to the manufacturer’s instruction. The Glucose uptake assay (Fluorometric, Direct Glucose) (Abcam, USA) was used to perform the glucose uptake assay. The glycolysis rate assay was measured with Glycolysis Assay [Extracellular acidification] (Abcam, USA).

#### Molecular dynamics simulations

All molecular dynamics simulations involved in this study were performed using Amber 22 software^68^ and the ff19SB force field.^73^ The solvent model employed was TIP3P, and appropriate counterions were added to neutralize the system. After system energy minimization, the temperature was gradually increased from 0 K to 310.15 K (37°C) within a heating time of no more than 500 ps. The system was then equilibrated under the NPT ensemble and maintained at 310.15 K (37°C). Finally, three independent 1000 ns atomistic molecular dynamics simulations were conducted under the isothermal-isobaric ensemble, using periodic boundary conditions. The SHAKE method was applied to constrain all covalent bonds involving hydrogen atoms. The calculation of long-range electrostatic interactions was performed using the PME method. The free energy of receptor-ligand binding was calculated by the MM/GBSA method using MMPBSA.py in Amber 22. The criteria for determining hydrogen bonds are as follows: (1) The distance between atom X and atom Y is less than 3.0 Å. (2) The angle between the X-H bond in the hydrogen bond donor and the Y atom in the hydrogen bond acceptor is less than 30°. The LCPO surface area of the ligand was calculated using the LCPO algorithm.^74^

#### Surface plasmon resonance (SPR) experiment

The full-length CDSs of *CgPk* and *CgPk*^*S11A*^ were amplified and inserted into pET-32a plasmid to express the 6 X His-tags at both N- and C-ends (Table S2). The binding activities of *Cg*PK and *Cg*PK^S11A^ toward PEP and ADP were measured by using BIAcore T200 SPR instrument (GE Healthcare, USA) and Series S Sensor Chip CM5 (GE Healthcare, USA). HBS-EP+ buffer (GE Healthcare, pH 7.5) was used as a running buffer. The anti-His tag antibody was immobilized onto the CM5 sensor chip surface according to the instructions of the Amine Coupling Kit (GE Healthcare, USA). *Cg*PK and *Cg*PK^S11A^ with His tag (20 μg/mL) were injected to bind the anti-His tag antibody with approximately 200 response units (RUs). The PEP (100 nM ∼ 1600 nM) and ADP (1.25 μM ∼ 10 μM) were diluted using PBS-P+ buffer (GE Healthcare, USA) and then injected into the wells and the flow wells were controlled at a flow rate of 30 μL/min for 120 s response. The bound proteins and PEP/ADP were washed with 10 mM glycine-HCl (pH 1.5; GE Healthcare, USA) at a flow rate of 30 μL/min for 60 s. The binding activity was quantitatively described as binding reaction value (response unit, RU), which was equal to the highest reaction value minus the baseline reaction value based on raw results on the SPR instrument. The kinetic curves were analyzed using BIAcore software on the SPR instrument, and the equilibrium dissociation constants (KD) were calculated using the BIAcore software 1:1 Langmuir binding model for fitting.

#### Prediction of kinase

The iGPS1.0 software was utilized for predicting potential kinases targeting the *Cg*IκBα S74 site. This prediction was based on the Short Linear Motif (SLM) theory, which focuses on the surrounding short linear motifs associated with the phosphorylation site (p-site), providing a high degree of specificity.^69^ For the analysis, *Homo sapiens* was chosen as the organism, with a threshold set to "medium" and the "interaction" parameter configured as "Exp./String".

#### Co-IP

Co-IP assays were conducted using the Flag-tag Protein IP Assay Kit with Magnetic Beads (Beyotime Biotechnology, China). The full-length CDSs of *CgPk* and *CgErk* were amplified and inserted into pCMV-N-Flag and pCMV-N-Myc plasmids for fusion with the tag (Table S2). After 36 hours of co-transfection into HEK293T cells, the Co-IP reaction was performed according to the manufacturer’s instructions. Briefly, the cells lysates were incubated with anti-Flag magnetic beads and Mouse IgG magnetic beads overnight. Following washing step, the reaction products were loaded onto 4∼20% SDS-PAGE gels (GenScript Biotech, China), and then the signals were obtained by western blotting.

#### Yeast two-hybrid assay

The full-length CDSs of *CgPk* and *CgErk* were amplified and inserted into pGBK-T7 and pGAD-T7 (MiaoLing Plasmid Platform, China), respectively (Table S2). Pairwise interactions were tested using GAL4 Yeast Two-Hybrid Media Kit (Coolaber, China). Briefly, each vector (bait and prey) was transformed in the Y2HGold yeast strain, and plated first on -Leu -Trp plates to allow selective growth of transformants. After 2-3 days, growth transformants were inoculated into (-Leu, -Trp) medium and allowed to grow overnight at 30°C with continuous shaking at 200 rpm. Subsequently, 10 μl of cell suspension, diluted in ddH_2_O to achieve an optical density (OD) of 0.5 and 1.0, was plated on selective plates (-Leu, -Trp), (-Leu, -Trp, -His3) and (-Leu, -Trp, -His3, -Ade2) and incubated for 2-3 days to assess the presence of interactions.

#### BiFC assay

The full-length CDSs of *CgPk* and *CgErk* were amplified and inserted into pBiFC-VN173 and pBiFC-VC155 plasmids (MiaoLing Plasmid Platform, China; Table S2). The Lipofectamine 3000 (Invitrogen, USA) was used to transfect these plasmids into HeLa cells (Procell Life Science & Technology, China). After incubation at 42°C for 2h, the fluorescence was imaged using a confocal microscope LSM710 (Carl Zeiss, Germany).

#### Subcellular localization

The full-length CDSs of *CgPk* and *CgErk* were amplified and inserted into pCMV-N-mCherry and pCMV-N-EGFP plasmids for fusion with the reporter gene, respectively (Beyotime Biotechnology, China). The cell culture, plasmid transfection and imaged for fluorescence as mentioned in the section “BiFC Assay”.

#### *In vivo* phosphorylation assay

Recombinant Flag-*CgPK* plasmid and its site-mutated variants, along with additional upstream plasmids such as Myc-*CgErk* plasmid, were co-transfected into HEK293T cells as above. After 36 h, the cells were lysed using a lysis buffer supplemented with protease and phosphatase inhibitors (Beyotime Biotechnology, China). The PK protein was subsequently purified using the anti-Flag magnetic beads as mentioned in the section “Co-IP”. The phosphorylation levels of the PK protein were then measured using the Phos-tag SDS-PAGE method. Phos-Tag™ was purchased from FUJIFILM Wako Pure Chemical Corporation (Japan) and used according to the manufacturer’s protocol. Briefly, gels used for Phos-tag SDS-PAGE consisted of a separating gel [8% (w/v) acrylamide, 375 mM Tris-HCl, pH 8.8, 50 μM Phos-tag acrylamide, 100 μM MnCl_2_, 0.1% SDS solution, 0.1% (v/v) N,N,N’,N’-tetramethylethylenediamine (TEMED), and 0.05% (w/v) ammonium persulfate (APS)], and a stacking gel [4.5% (w/v) acrylamide, 125 mM Tris-HCl, pH 6.8, 0.1% SDS solution, 0.1% (v/v) N,N,N’,N’-tetramethylethylenediamine (TEMED), and 0.05% (w/v) ammonium persulfate (APS)]. Electrophoresis was conducted at a constant current of ≤30 mA/gel with the running buffer [25 mM Tris-base, 192 mM glycine, and 0.1% (w/v) SDS]. For western blotting analysis, gel was washed using wash buffer [25 mM Tris, 192 mM glycine, 0.1% (v/v) SDS, 10 mM EDTA] for 10 min three times to remove metal ions, followed by one wash without EDTA for 10 min. Then, the samples were electroblotted to the polyvinylidene difluoride (PVDF) membrane (Millipore, USA) for subsequent western blotting.

#### *In vitro* kinase activity assay

The full-length CDSs of *CgPk* and *CgErk1* and their site-mutated variants, were amplified and inserted into pET-32a plasmid to express the 6 X His-tags at both N- and C-ends (Table S2). The purified proteins were used to test whether recombinant *Cg*ERK could phosphorylate *Cg*PK, as well as to evaluate the influence of phosphorylation at the T187 and Y189 sites of *Cg*ERK12 on its ability to phosphorylate *Cg*PK. The wild type and S11A mutant of *Cg*PK was used as substrate. The kinase activity assay was performed in 20 μl kinase buffer containing 25 mM Tris-HCl, pH 7.5, 5 mM beta-glycerophosphate, 2 mM dithiothreitol (DTT), 0.1 mM Na_3_VO_4_, 10 mM MgCl_2_, 20mM ATP (CST, USA), 1 μg substrate protein and 1 μg kinase protein for 30 min at 30°C. And the reactions were stopped with SDS loading buffer. The phosphorylation levels of the *Cg*PK protein were then measured using western blotting with anti-phosphoserine/threonine antibody (ECM, PP2551; 1:1,000 dilution).

#### *In vivo* upstream regulators validation

The full-length CDSs of *CgPk* and its site-mutated variants, along with additional upstream plasmids such as Myc-*CgErk* plasmid, were co-transfected into HEK293T cells as above (Table S2). After 36 hours, the PK enzyme activity, PA and ATP content was measured as above. Additionally, the ERK inhibitor SCH772984 (HY- 50846; MCE, USA) in DMSO was prediluted with complete media and then added to the cells to reach a final concentration of 50, 100, 200 and 300 nM for 8 hours to inhibit the *Cg*ERK activity.

#### Raw data Reanalysis

The transcriptomic and protein phosphorylation omics data for *C. gigas* and *C. angulata* under 12 h of heat stress were downloaded from our previous study.^8^^,^^38^ The clean reads of RNA-Seq were aligned to the oyster genome (GenBank accession no. GCA_011032805.1)^75^ using HISAT2 v2.1.0.^76^ The HTSeq v0.6.0 tool was used to count the reads for each gene in each sample to quantify gene expression.^77^

#### Western blotting

The samples from oysters and cells were extracted using Cell lysis buffer for Western and IP (Beyotime Biotechnology, China) and M-PER™ Mammalian Protein Extraction Reagent containing protease inhibitor (Thermo Fisher Scientific, USA) supplemented with protease and phosphatase inhibitors (Beyotime Biotechnology, China), respectively. The supernatant protein was subjected to denaturation at 100°C for 10 min after the addition of 4X protein loading buffer (GenScript Biotech, China). Then, the proteins were transferred onto 0.45 nm pore polyvinylidene fluoride (PVDF) membrane (Millipore, USA) using an eBlot™ L1 wet transfer (GenScript Biotech, China). Membranes were blocked and incubated with primary antibodies and secondary antibodies using eZwest Lite Automated Western Device (GenScript Biotech, China). Membranes were then incubated with Omni-ECL™Femto Light Chemiluminescence Kit (Epizyme, China) and captured using the Molecular Imager® Gel Doc™ XR System (Bio-Rad, USA). The antibodies used were as follows: Flag-tag (ZENBIO, 390002), Myc-tag (ZENBIO, 390003), beta Actin (ZENBIO, 200068-8F10), His-tag (ZENBIO, 230001), ERK1/ERK2 (ABclonal, A16686), Phospho-ERK1-T202/Y204 + ERK2-T185/Y187 (ABclonal, AP0472), MAP2K1/MAP2K2 (ABclonal, A24394), Phospho-MAP2K1-S217/MAP2K2-S221 (ABclonal, AP0209), MAP3K1 (ABclonal, A21490), HRP-labeled Goat Anti-Mouse/Rabbit IgG(H+ +L) (Epizyme, LF101) and HRP-labeled Goat Anti-Mouse/Rabbit IgG(H+L) (Epizyme, LF102).

### Quantification and statistical analysis

All statistical analyses were performed using GraphPad Prism version 8.0.2 for Windows. After confirming the normality of the distributions using the Shapiro–Wilk test and homogeneity of variance using Bartlett’s test, data were analyzed with the two-tailed unpaired Student’s t-test, one-way analysis of variance (ANOVA) and two-way ANOVA followed by Tukey’s multiple comparisons test. Data are shown as the means ± SD, and the number of replicates (n) are denoted in the corresponding figure legends. Significant differences between groups were marked with “^∗^” for *p*< 0.05, “^∗∗^” for *p*<0.01, “^∗∗∗^” for *p*<0.001 and “^∗∗∗∗^” for *p*<0.0001.
