# Supplementary material for: MAPK/ERK-PK(Ser11) pathway regulates divergent thermal metabolism of two congeneric oyster species
Source: iScience. 2024 Jun 20;27(7):110321. doi: 10.1016/j.isci.2024.110321 (PMC11269933; doi:10.1016/j.isci.2024.110321)

iScience, Volume *27*

Supplemental information

MAPK/ERK-PK(Ser11) pathway regulates divergent thermal metabolism of two congeneric oyster species

Chaogang Wang, Mingyang Du, Zhuxiang Jiang, Rihao Cong, Wei Wang, Taiping Zhang, Jincheng Chen, Guofan Zhang, and Li Li

Supplementary File

Table S1 Vertebrates and invertebrates PK protein sequences analyzed in this study

Phylum	Specie	Name	GenBank accession No.	
			CDS	Protein
Chordata	*Homo sapiens*	*Hm* PKM	NM_001206796.3	NP_001193725.1
		Hm PKLR	NM_000298.6	NP_000289.1
	Mus musculus	*Mm* PKM	NM_001253883.2	NP_001240812.1
		Mm PKLR	NM_013631.2	NP_038659.2
Echinodermata	*Strongylocentrotus purpuratus*	*Sp* PK	XM_031000414.1	XP_030856274.1
	Asterias rubens	*Ar* PK	XM_033768307.1	XP_033624198.1
	Acanthaster planci	*Ap* PK	XM_022249863.1	XP_022105555.1
	Patiria miniata	*Pm* PK	XM_038222719.1	XP_038078647.1
	Apostichopus japonicus	Aj PK	KT779930.1	ALJ77068.1
Arthropoda	*Drosophila melanogaster*	*Dm* PK	NM_165152.2	NP_723960.1
	Vanessa atalanta	*Va* PK	XM_047672986.1	XP_047528942.1
	Nymphalis io	*Ni* PK	XM_050487728.1	XP_050343685.1
Nematodes	*Caenorhabditis elegans*	Ce PK	NM_060057.7	NP_492458.1
Mollusca	*Crassostrea gigas* (GCA_011032805.1)	*Cg* PK	Genome BLAST	Genome BLAST
	Crassostrea ariakensis (GCA_020567875.1)	*Ca* PK	Genome BLAST	Genome BLAST
	Crassostrea virginica	*Cv* PK	XM_022456022.1	XP_022311730.1
	Ostrea edulis	*Oe* PK	XM_048893154.1	XP_048749111.1
	Patella vulgata	*Pv* PK	XM_050540378.1	XP_050396335.1
	Dreissena polymorpha	*Dp* PK	-	KAH3746712.1
	Pomacea canaliculata	*Pc* PK	XM_025225728.1	XP_025081513.1
	Gigantopelta aegis	*Ga* PK	XP_041356453.1	XP_041356453.1
	Haliotis rufescens	*Hr* PK	XM_046508536.2	XP_046364492.1
	Haliotis fulgens	*Hf* PK	MH220522.1	AXR98604.1
	Haliotis rubra	Hru PK	XM_046688104.1	XP_046544060.1
	Biomphalaria glabrata	*Bg* PK	XP_013085036.1	XP_013085036.1
	Lottia gigantea	*Lg* PK	XM_009048490.1	XP_009046738.1
Cnidaria	*Pocillopora*	*Pd* PK	XM_027196829.1	XP_027052630.1

	damicornis			
	Acropora millepora	*Am* PK	XM_029356235.2	XP_029212068.1
	Orbicella faveolata	*Of* PK	XM_020769753.1	XP_020625412.1
	Actinia tenebrosa	*At* PK	XM_031708778.1	XP_031564638.1
Porifera	*Amphimedon queenslandica*	*Aq* PK	XM_003385723.3	XP_003385771.1

Table S2 Primer sequences for plasmid construction.

Name	Primer Sequence (5′-3′)
**Flag-PK**	F: ctgtacaagggatccaagcttATGGCACAGCACCTTGCAA<br>R: atcgaattcctgcagaagcttTCAAAAGAACTTGACATCGTCCTT
**Flag-*Cg*PKS11A**	F: CgcTGGGTTTAACGTGAAGGAGATGGAGAGAGA<br>R: TTCACGTTAAACCCAgcGCCTGGTGTCTTTGCAAGGTG
**Flag-*Cg*PKS11D**	F: ACCAGGCgaTGGGTTTAACGTGAAGGAGATGGA<br>R: TTAAACCCAtcGCCTGGTGTCTTTGCAAGGTGC
**32a-PK**	F: gctgatatcggatccgaattcATGGCACAGCACCTTGCAA<br>R: ttgtcgacggagctcgaattcAAAGAACTTGACATCGTCCTTTGA
**Myc-ERK1/2**	F: tccaagcttctgcaggaattcATGGCGAGCGGGGATAAA<br>R: tctgtcgacgatatcgaattcTTATAAATCAGCAATTCCCATTTTCT
**155-ERK1/2**	F: accgagatctctcgaggtaccATGGCGAGCGGGGATAAA<br>R: tttgcacgccggacgggtaccTAAATCAGCAATTCCCATTTTCTTC
**AD-ERK1/2**	F: gtaccagattacgctcatatgATGGCGAGCGGGGATAAA<br>R: actggcctccatggccatatgTTATAAATCAGCAATTCCCATTTTCT
**BD-PK**	F: TCAGAGGAGGACCTGCATATGATGGCACAGCACCTTGCAA<br>R: TTCGGCCTCCATGGCCATATGTCAAAAGAACTTGACATCGTCCTT
**173-PK**	F: CTGATATCGGTACCAGTCGACATGGCACAGCACCTTGCAA<br>R: GATGGATCTTCTAGAGTCGACAAAGAACTTGACATCGTCCTTTGA
**mCherry-PK**	F: ctgtacaagggatccaagcttATGGCACAGCACCTTGCAA<br>R: atcgaattcctgcagaagcttTCAAAAGAACTTGACATCGTCCTT
**32a-ERK1/2**	F: gacaaggccatggctgatatcATGGCGAGCGGGGATAAA<br>R: ctcgaattcggatccgatatcTTATAAATCAGCAATTCCCATTTTCT
**EGFP-ERK1/2**	F: ctgtacaagggatccaagcttATGGCGAGCGGGGATAAA<br>R: atcgaattcctgcagaagcttTTATAAATCAGCAATTCCCATTTTCT
**ERK1/2T187DY189E**	F: TTTGgacgagtttGTCGCAACAAGATGGTACAGAGC<br>R: CGACaaactcgtcCAAAAATCCAGTGTGGTCATGATT
**ERK1/2T187AY189F**	F: TTTGgcagagtttGTCGCAACAAGATGGTACAGAGC<br>R: CGACaaactctgcCAAAAATCCAGTGTGGTCATGATT
**His-MAP2K1**	F: tccaagcttctgcaggaattcATGAGTGAACATTCTGTCACATCAAA<br>R: tctgtcgacgatatcgaattcTTAGCTGTTCTTCTGTTCCGGTT

Table S3 The results of prediction of kinase.

ID	Position	Code	Protein description	Matched ID	Gene name	Kinase ID	Kinase Name	Interaction	Predictor	Score	Cutoff

g02711	11	S	HLAKTPGSGFNVKEM	P14618	PKM2	Q13418	ILK	String	TKL/MLK	1.25	0
g02712	11	S	HLAKTPGSGFNVKEM	P14618	PKM2	Q12852	DLK	String	TKL/MLK	1.25	0
g02713	11	S	HLAKTPGSGFNVKEM	P14618	PKM2	Q59H18	HH498	String	TKL/MLK	1.25	0
g02714	11	S	HLAKTPGSGFNVKEM	P14618	PKM2	B8ZZU2	ZAK	String	TKL/MLK	1.25	0
g02715	11	S	HLAKTPGSGFNVKEM	P14618	PKM2	P80192	MLK1	String	TKL/MLK	1.25	0
g02716	11	S	HLAKTPGSGFNVKEM	P14618	PKM2	P19525	PKR	String	Other/PEK	0.81	0
g02717	11	S	HLAKTPGSGFNVKEM	P14618	PKM2	B2RCU9	PEK	String	Other/PEK	0.81	0
g02718	11	S	HLAKTPGSGFNVKEM	P14618	PKM2	P22612	PKACg	String	AGC/PKA	0.59	0
g02719	11	S	HLAKTPGSGFNVKEM	P14618	PKM2	P17612	PKACa	String	AGC/PKA	0.59	0
g02720	11	S	HLAKTPGSGFNVKEM	P14618	PKM2	P51817	PRKX	String	AGC/PKA	0.59	0
g02721	11	S	HLAKTPGSGFNVKEM	P14618	PKM2	P22694	PKACb	String	AGC/PKA	0.59	0
g02722	11	S	HLAKTPGSGFNVKEM	P14618	PKM2	P27361	Erk1	String	CMGC/MAPK	0.58	0
g02723	11	S	HLAKTPGSGFNVKEM	P14618	PKM2	Q13164	Erk5	String	CMGC/MAPK	0.58	0
g02724	11	S	HLAKTPGSGFNVKEM	P14618	PKM2	Q16659	Erk3	String	CMGC/MAPK	0.58	0
g02725	11	S	HLAKTPGSGFNVKEM	P14618	PKM2	A8MTD8	JNK3	String	CMGC/MAPK	0.58	0
g02726	11	S	HLAKTPGSGFNVKEM	P14618	PKM2	D3DX92	JNK1	String	CMGC/MAPK	0.58	0
g02727	11	S	HLAKTPGSGFNVKEM	P14618	PKM2	Q15759	p38b	String	CMGC/MAPK	0.58	0
g02728	11	S	HLAKTPGSGFNVKEM	P14618	PKM2	P45984	JNK2	String	CMGC/MAPK	0.58	0
g02729	11	S	HLAKTPGSGFNVKEM	P14618	PKM2	P28482	Erk2	String	CMGC/MAPK	0.58	0
g02711	11	S	HLAKTPGSGFNVKEM	P14618	PKM2	Q59H18	HH498	String	TKL/MLK	1.25	0
g02712	11	S	HLAKTPGSGFNVKEM	P14618	PKM2	B8ZZU2	ZAK	String	TKL/MLK	1.25	0
g02713	11	S	HLAKTPGSGFNVKEM	P14618	PKM2	P80192	MLK1	String	TKL/MLK	1.25	0
g02714	11	S	HLAKTPGSGFNVKEM	P14618	PKM2	P19525	PKR	String	Other/PEK	0.81	0
g02715	11	S	HLAKTPGSGFNVKEM	P14618	PKM2	B2RCU9	PEK	String	Other/PEK	0.81	0

g02716	11	S	HLAKTPGSGFNVKEM	P14618	PKM2	P22612	PKACg	String	AGC/PKA	0.59	0
g02717	11	S	HLAKTPGSGFNVKEM	P14618	PKM2	P17612	PKACa	String	AGC/PKA	0.59	0
g02718	11	S	HLAKTPGSGFNVKEM	P14618	PKM2	P51817	PRKX	String	AGC/PKA	0.59	0
g02719	11	S	HLAKTPGSGFNVKEM	P14618	PKM2	P22694	PKACb	String	AGC/PKA	0.59	0
g02720	11	S	HLAKTPGSGFNVKEM	P14618	PKM2	P27361	Erk1	String	CMGC/MAPK	0.58	0
g02721	11	S	HLAKTPGSGFNVKEM	P14618	PKM2	Q13164	Erk5	String	CMGC/MAPK	0.58	0
g02722	11	S	HLAKTPGSGFNVKEM	P14618	PKM2	Q16659	Erk3	String	CMGC/MAPK	0.58	0
g02723	11	S	HLAKTPGSGFNVKEM	P14618	PKM2	A8MTD8	JNK3	String	CMGC/MAPK	0.58	0
g02724	11	S	HLAKTPGSGFNVKEM	P14618	PKM2	D3DX92	JNK1	String	CMGC/MAPK	0.58	0
g02725	11	S	HLAKTPGSGFNVKEM	P14618	PKM2	Q15759	p38b	String	CMGC/MAPK	0.58	0
g02726	11	S	HLAKTPGSGFNVKEM	P14618	PKM2	P45984	JNK2	String	CMGC/MAPK	0.58	0
g02727	11	S	HLAKTPGSGFNVKEM	P14618	PKM2	P28482	Erk2	String	CMGC/MAPK	0.58	0

Fig. S1 Phylogenetic tree of oyster, human, mouse and zebrafish ERK gene family. The tree was constructed with the maximum likelihood (ML) method using PhyloSuite. Bootstrap support values are indicated by sizes on nodes of phylogenetic tree. The *Cg*ERK1/2 is marked with red bold line. Different colors indicate different categories of ERK subfamilies. Cg, *Crassostrea gigas*; Hm, *Homo sapiens*; Mm, *Mus musculus*; Dr, *Danio rerio*. The accession numbers were as follows: *Hm* ERK1, NP_002737.2; *Hm* ERK2, NP_002736.3; *Hm* ERK3, NP_002739.1; *Hm* ERK4, NP_002738.2; *Hm* ERK5, NP_002740.2; *Hm* ERK6, NP_002960.2; *Hm* ERK7, NP_620590.2; *Mm* ERK1, NP_036082.1; *Mm* ERK2, NP_001033752.1; *Mm* ERK3, NP_056621.4; *Mm* ERK4, NP_766220.2; *Mm* ERK5, NP_001277963.1; *Mm* ERK6, NP_001389948.1; *Mm* ERK7, NP_808590.1; *Dr* ERK1, NP_958915.1; *Dr* ERK3, NP_001039017.1; *Dr* ERK4, NP_998638.1; *Dr* ERK5, NP_001013469.2; *Dr* ERK6, NP_571482.1; *Dr* ERK7, XP_009292731.1. The sequences of *Cg*ERK1/2 (Cg03498) gene in oyster were obtained from *Crassostrea gigas* genome blast (GCA_011032805.1).

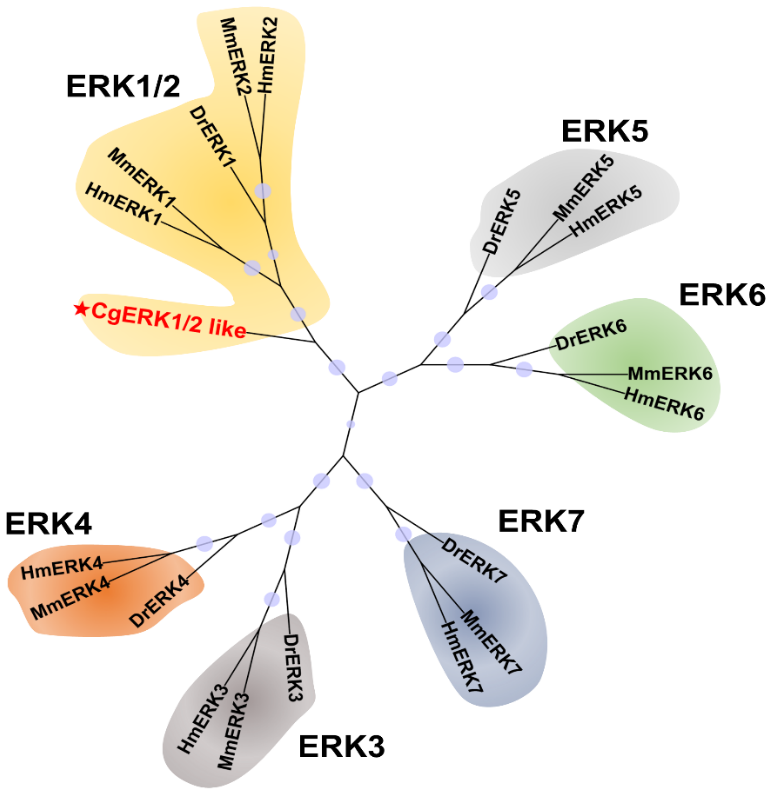

Supplement: Document S1. Figure S1 and Tables S1–S3 [file mmc1.pdf]
